# P-1900. Insights into Antimicrobial and Interprofessional Reasoning Processes Among Medical and Pharmacy Residents

**DOI:** 10.1093/ofid/ofaf695.2069

**Published:** 2026-01-11

**Authors:** Katherine Gruenberg, Emily Abdoler, Conan MacDougall

**Affiliations:** UCSF School of Pharmacy, San Francisco, CA; University of Michigan, Ann Arbor, MI; University of California San Francisco, San Francisco, CA

## Abstract

**Background:**

Management reasoning, the process underlying treatment selection, is central to the work of physicians and pharmacists. However, their roles in this process are both different and also often complementary. Interprofessional practice is especially important in the realm of antimicrobials, where healthcare professionals share responsibility for antimicrobial stewardship. Despite its importance, little is known about how professionals approach antimicrobial management reasoning (AMR). Our previous work uncovered a broad, multifactorial process that underlies both physician and pharmacist AMR. To further investigate this gap, we conducted a study to characterize AMR processes among pharmacy and internal medicine residents at two academic institutions.

**Table 1:**
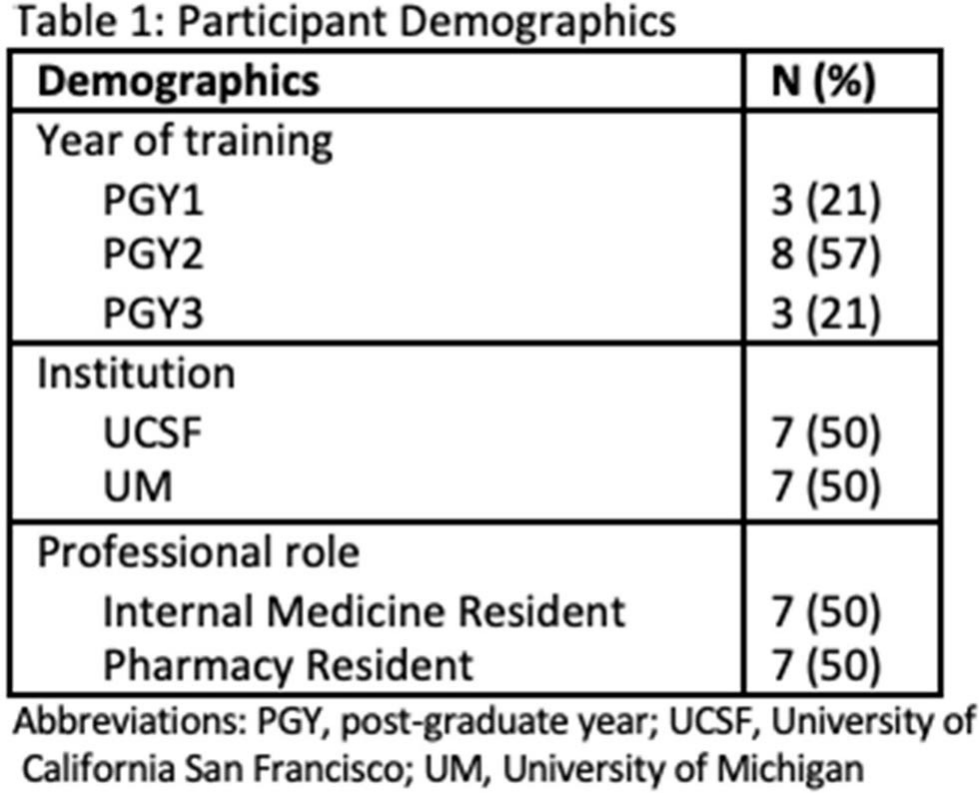
Participant Demographics

**Table 2:**
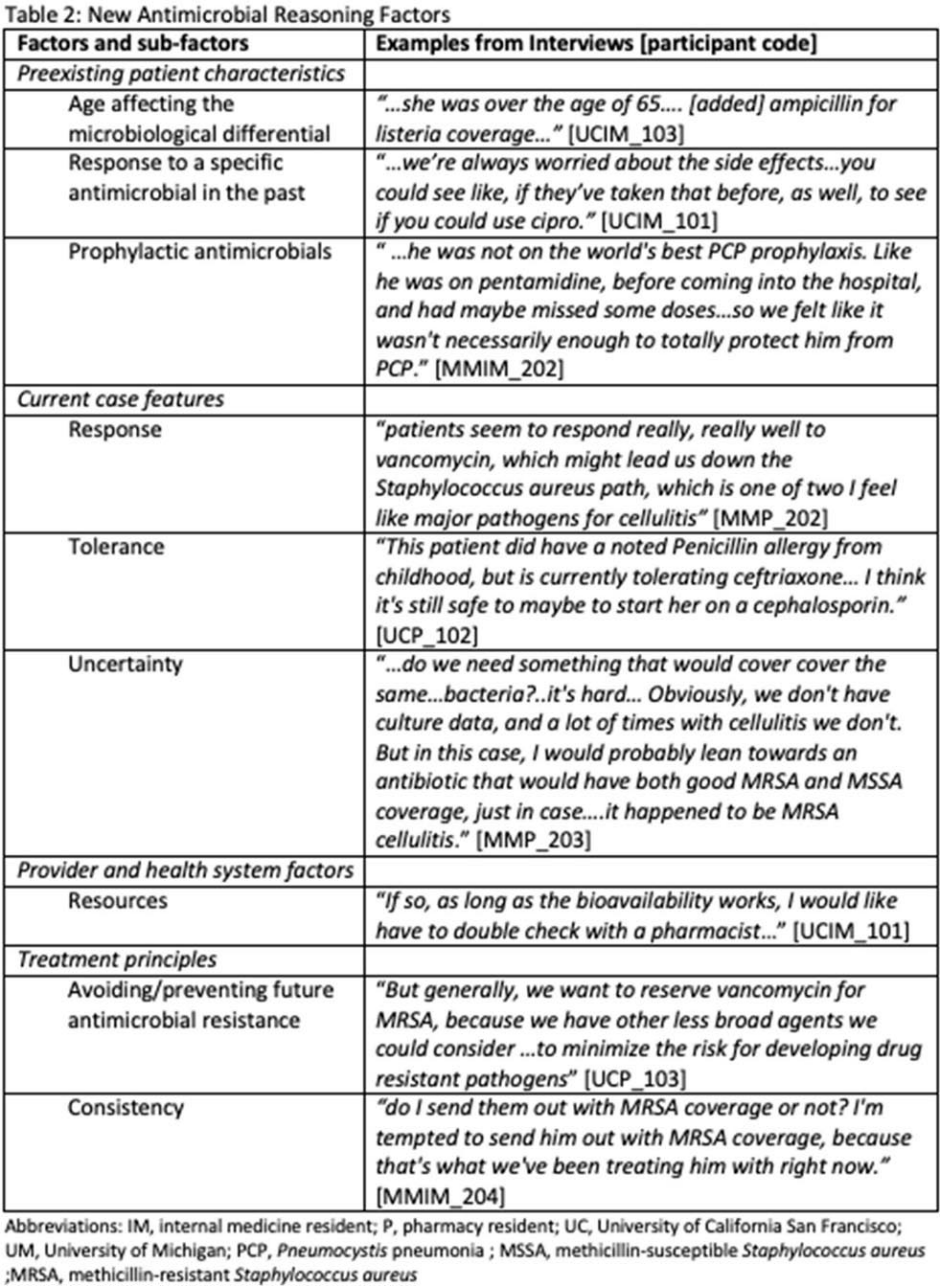
New Antimicrobial Reasoning Factors

**Methods:**

Fourteen residents participated in semi-structured interviews using clinical vignettes (pneumonia, cellulitis, urosepsis) and reasoning-mapping exercises. Transcripts were thematically analyzed using a sensitizing framework from our prior research and the Interprofessional Education Collaborative (IPEC) competencies.

**Table 3:**
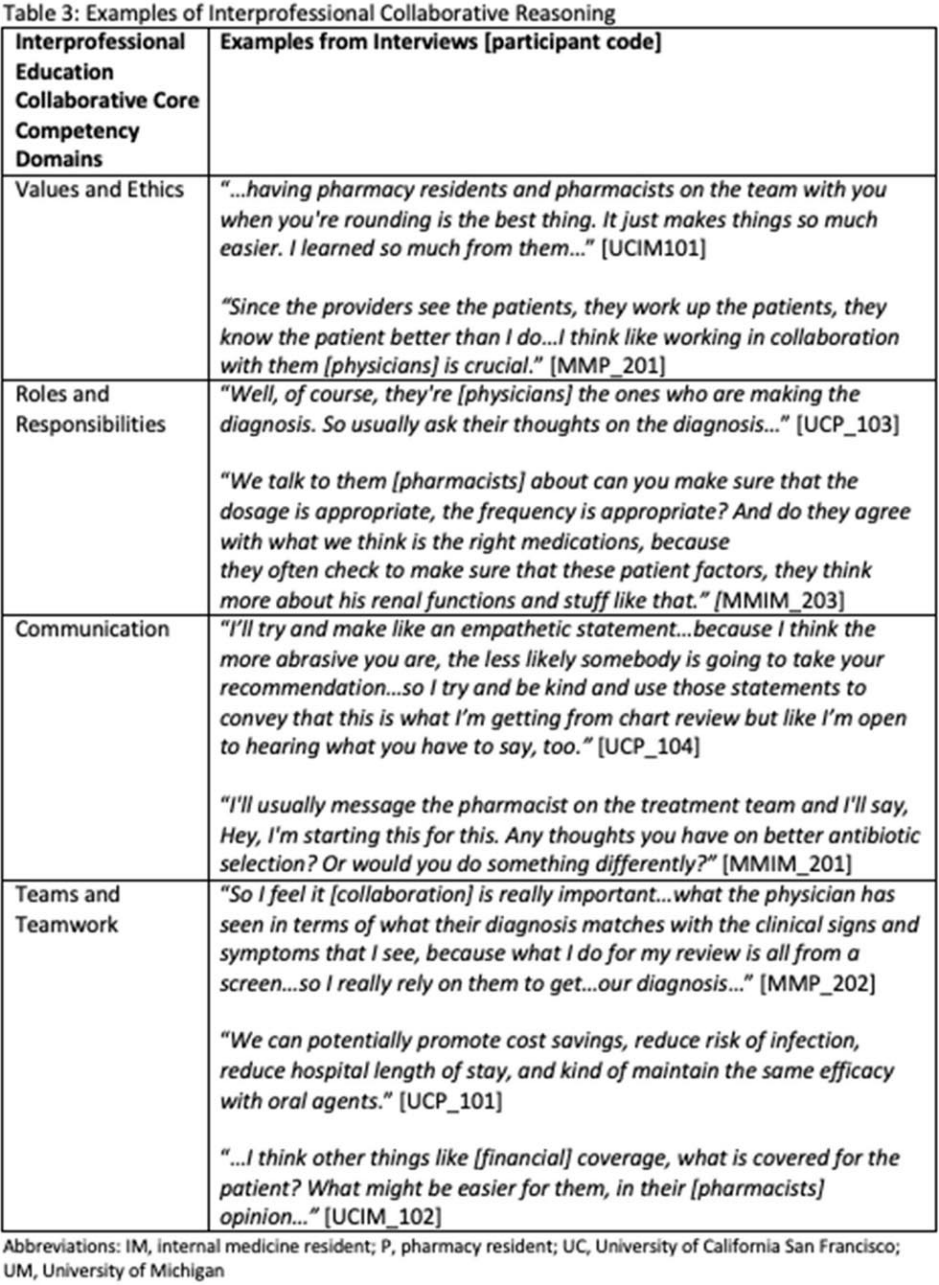
Examples of Interprofessional Collaborative Reasoning

**Results:**

Participants generally followed a three-step reasoning process—naming the syndrome, delineating pathogens, and antimicrobial selection—similar to experienced clinicians. However, residents demonstrated an expanded script that incorporated new insights related to patient characteristics, case features, and treatment principles. Respondents described collaborative AMR that spanned each IPEC Domain. Examples included participants valuing the skills of their interprofessional colleagues, collaborating and incorporating complementary expertise, using communication techniques to optimize team dynamics, and making team-based decisions.

**Conclusion:**

These findings highlight how trainees conceptualize AMR and interprofessional practice around antibiotic selection, expanding our understanding of interprofessional management reasoning generally and offering insights into potential educational interventions in antimicrobial stewardship. Our next study will investigate real-time collaborative AMR between medical and pharmacy residents to optimize interprofessional training and stewardship outcomes.

**Disclosures:**

All Authors: No reported disclosures

